# Hem-o-Lok® clip migration after robotic prostatectomy: PRISMA-guided review with two illustrative cases

**DOI:** 10.1080/20905998.2026.2623583

**Published:** 2026-02-16

**Authors:** C. P. Leiberman, X. W. J. Lee, T. Amer, S. K. Nalagatla

**Affiliations:** a Department of Urology, University Hospital Monklands, Airdrie, UK; b Department of Urology, Victoria Hospital, Fife, UK

**Keywords:** Hem-o-Lok migration, robotic-assisted laparoscopic prostatectomy, bladder stone, lower urinary tract symptoms

## Abstract

Hem-o-Lok® polymer clips are widely used during robot-assisted radical prostatectomy. Although generally safe, delayed intravesical migration is an under-recognized source of lower urinary tract symptoms, haematuria and bladder stone formation. We report two illustrative cases – one with large bladder calculus formed around an eroded clip and another with spontaneous urethral passage – then synthesize the contemporary literature using a PRISMA-guided search. Across > 100 reported patients, time to presentation ranges from 1 to 252 months. The commonest presentations are LUTS, haematuria and recurrent infection; most cases are diagnosed by cystoscopy (often with confirmatory CT) and managed endoscopically with clip extraction ± laser lithotripsy, with open removal reserved for inaccessible or embedded clips. Proposed mechanisms include clip placement close to the vesicourethral anastomosis, devitalized tissue at the anastomosis, urine leak and radiotherapy-related impaired healing. Preventive strategies center on minimizing clip use near the anastomosis, meticulous retrieval of loose clips, judicious energy use at the apex/bladder neck, and considering alternatives (suture ligation or vessel-sealing devices) where appropriate. Increased awareness can shorten time to diagnosis and reduce morbidity for post-prostatectomy patients presenting with non-specific urinary symptoms years after surgery.

## Introduction

Robot-assisted laparoscopic prostatectomy (RALP) is a common and effective surgical approach for the management of localised prostate cancer, offering improved precision and postoperative recovery [1,2]. Hem-o-Lok® clips (HOLC), non-absorbable polymer locking clips introduced in 1999, are extensively used during RALP for vascular control and anastomotic support due to their efficiency and ease of use [3–6].

While generally considered safe, rare but important complications associated with HOLC have been increasingly recognised. One such complication is delayed intravesical migration, which may result in bladder stone formation, lower urinary tract symptoms (LUTS), and urethrovesical anastomotic complications [3,7]. Despite growing awareness, the true incidence, mechanisms, and optimal management strategies remain poorly characterised.

In this paper, we present two patients who developed delayed complications from intravesical HOLC migration years after RALP. We supplement these cases with a focused review of the literature to synthesise common patterns in clinical presentation, diagnostic approaches, and management. This work aims to raise awareness and to reduce diagnostic delays and associated morbidity.

## Methods

A structured literature search was conducted in accordance with the PRISMA 2020 guidelines. The following databases were searched from inception to 31 August 2025: PubMed, EMBASE (Ovid), Scopus, Cochrane Library, CINAHL (EBSCOhost), and Google Scholar. The search strategy included combinations of keywords and subject headings related to Hem-o-Lok clips, robotic prostatectomy, and clip migration or associated complications.

Boolean operators and truncation were used to optimise sensitivity. Key search terms included: ‘Hem-o-Lok’, ‘polymer clip’, ‘robotic prostatectomy’, ‘robot-assisted radical prostatectomy’, ‘clip migration’, ‘foreign body’, and ‘bladder stone’.

### Eligibility criteria

Studies were included if they met the following criteria:

- Reported Hem-o-Lok or polymer clip migration following radical prostatectomy, including robotic-assisted approaches, where clinical presentation and management were clearly described.
- Provided clinical detail on presentation, diagnostic findings, timing, management, or outcome

#### Exclusion criteria

Studies not involving robotic prostatectomy

- Review articles, letters, editorials, animal studies, or laboratory-based work
- Studies not reporting clip migration or lacking sufficient clinical information

### Study selection

Search results were imported into reference management software (Rayyan) and duplicates were removed. Titles and abstracts were screened for relevance. Full texts of potentially eligible studies were then reviewed against inclusion criteria.

### Data extraction and synthesis

Variables included author, year, number of patients, time to presentation, clinical presentation, stone formation, and management. Due to heterogeneity in reporting and the case-based nature of the literature, a narrative synthesis was performed, and results summarised in Table 1.

**Table 1.** Summary of reported cases of Hem-o-Lok clip migration following robotic-assisted laparoscopic prostatectomy.

Author (Year)	Patients	Interval from RALP (Months)	Presentation	Stone Formation	Management
Allaparthi [8]	1	18	LUTS	✓	Holmium laser Cystolitholapaxy
Blumenthal et al. [9]	4	11 [mean]	LUTS, Haematuria, Pyuria	x	Cystoscopic removal
Banks et al. [10]	1	10	Bladder spasms	✓	Cystolitholapaxy and removal
Moser & Narepalem [11]	2	3 - 4	BNC, Voiding dysfunction	x	Rigid cystoscopy, clip removal, dilation to 24F
Yi et al. [12]	2	2 - 4	Bladder neck contracture	x	Cystoscopic clip removal + urethral dilatation
Arias et al. [13]	1	n/a	n/a	n/a	Endoscopic removal
Shin et al. [1]	1	3	Dysuria, Weak stream	x	Cystoscopic clip removal
Cormio et al.[14]	1	3	Acute urinary retention	x	cold-knife incision, Endoscopic removal, Bladder neck resection
Parihar et al. [15]	1	7.1	Voiding difficulty (BNC symptoms)	x	Cold-knife bladder neck incision + ISC
Swaroop et al. [16]	2	3 - 12	LUTS: urgency, frequency, dribbling	✓	Cystoscopic holmium laser lithotripsy and clip removal
Yu et al. [17]	6	2 – 81	Type I: LUTS; Type II: Haematuria; Type III: Spontaneous clip expulsion	✓	Cystoscopic removal, holmium laser, dilation for BNC
Bientinesi et al. [18]	1	12	Dysuria, Nocturia, Frequency, and Haematuria	✓	Endoscopic removal
Jaeger et al. [19]	17*	1 – 252*	LUTS, Perineal pain, Haematuria, UTI, BNC*	n/a	Endoscopic removal*
Wong & Jain [20]	1	26	Urinary retention, recurrent UTIs, incontinence	✓	Laser lithotripsy with removal of 2 eroded Hem-o-Lok clips
Aoki et al [21]	1	3	Frequency, Haematuria	✓	Cystolitholapaxy and removal
Turini et al. [22]	8	21 [mean]	LUTS, UTI, Haematuria	✓	Endoscopic removal (laser, litholapaxy, forceps)
Lemura et al. [23]	4	2 - 26	LUTS, Perineal pain, Haematuria, Dysuria	n/a	Holmium laser lithotripsy, Spontaneous expulsion, Conservative
Chen et al. [24]	1	60	Haematuria	✓	Laser fragmentation, transurethral resection
Üçpınar et al. [2]	2	23 - 24	LUTS: dysuria, frequency, incomplete voiding	✓	Holmium laser lithotripsy + clip removal
Zhu et al. [25]	26	1 - 37	LUTS, Urinary retention	✓	Cystoscopy + removal of foreign body and/or lithotripsy
Ohyama et al. [26]	3	30 - 63	LUTS, Dysuria	✓	Holmium laser lithotripsy and removal
Kyzlasov et al. [27]	1	24	Dysuria, LUTS, Haematuria	✓	Transurethral laser Cystolitholapaxy, vaporisation of neck scar tissue, removal of clip
Chen et al. [28]	1	6	Dysuria, LUTs	n/a	BNI and Endoscopic removal
Deen et al. [29]	1	132	Recurrent UTI, LUTS, Visible haematuria	✓	Cystolitholapaxy using Swiss LithoClast® Trilogy; clip removed
Hakamada et al. [30]	1	28	Haematuria, Dysuria	✓	Holmium laser Cystolitholapaxy + clip removal
Nistiana et al. [3]	1	120	LUTS: dysuria, frequency	✓	Cystoscopic lithotripsy and clip extraction
Ahmad et al. [31]	1	120	LUTS, Haematuria, Dysuria	✓	Cystolitholapaxy and stone removal
Singh et al. [32]	9	1 – 36	LUTS, Haematuria, BNC	✓	Cystoscopy ± BNI; clip removal, bladder neck resection, self-calibration
Kotov et al. [33]	1	8	Frequency, Haematuria	✓	Transurethral laser lithotripsy and foreign body extraction
Paladini et al. [34]	1	n/a	BNC	x	Endoscopic holmium laser clip removal
Albadawe et al. [35]	1	15	LUTS, rUTIs	✓	Thulium laser lithotripsy and removal
Vega et al. [36]	1	3	LUTS: voiding difficulty, leakage	x	Panendoscopic forceps removal (FlexiBite-Medi™)
Bogomolov et al. [5]	3	3 to 50	Dysuria, Haematuria	✓	Spontaneous expulsion, Laser Cystolitholapaxy
Onal et al. [4]	5	27.8 [mean]	LUTS, Persistent dysuria	n/a	Single Cystoscopic intervention

BNI = bladder neck incision; ISC = intermittent self-catheterisation; LUTS = lower urinary tract symptoms; UTI = urinary tract infection; BNC = bladder neck contracture. ‘Stone Formation’ refers to radiological or endoscopic evidence of bladder calculus at the time of presentation. ‘n/a’ indicates data not reported.

*Jaeger et al (2015) did not differentiate between RP vs RALP.

A PRISMA flow diagram outlining the study selection process is presented in Figure 1.

**Figure 1.**
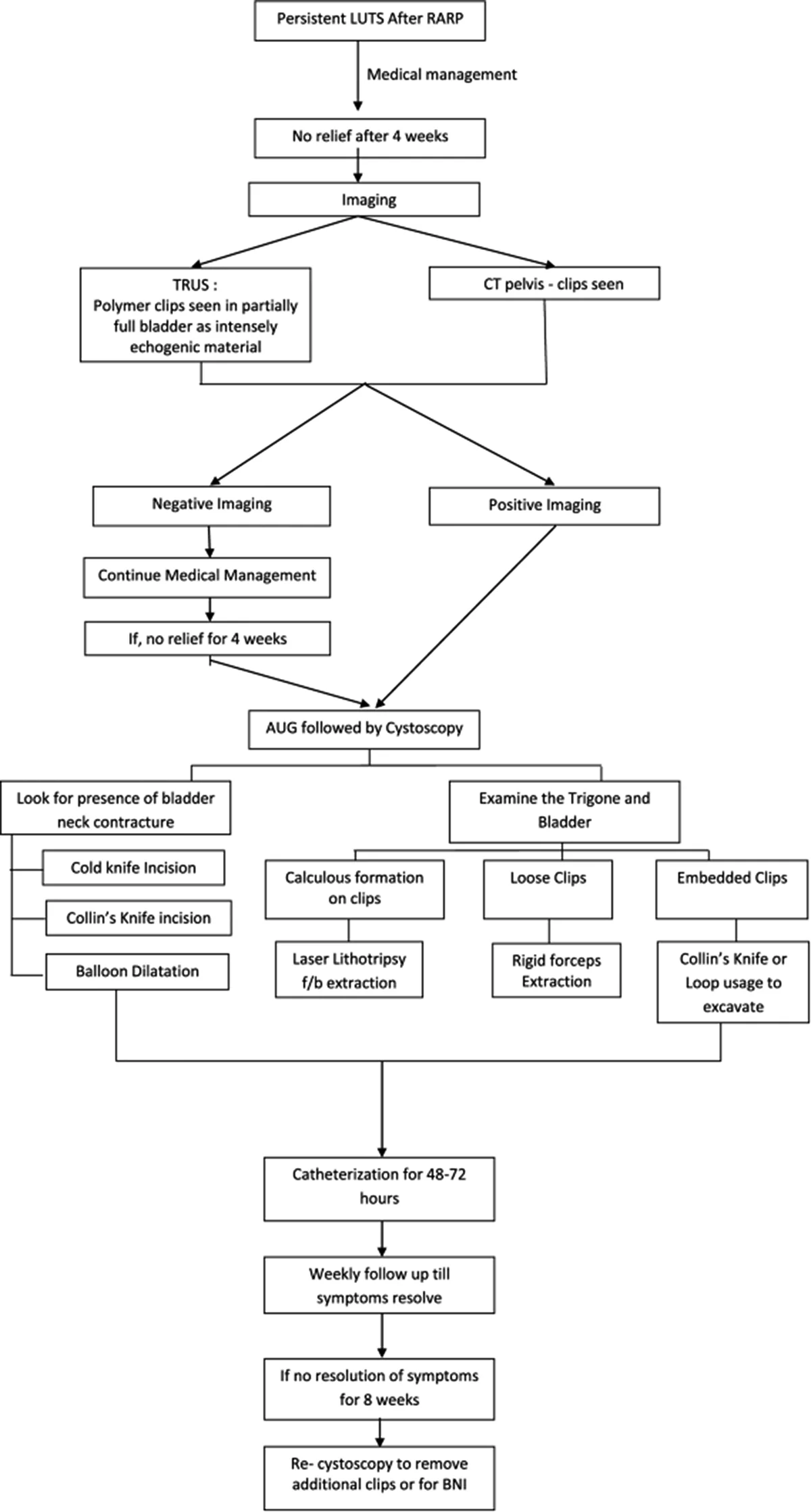
PRISMA 2020 flow diagram summarising study selection process [37].

## Results

A total of 34 publications were identified reporting HOLC migration following RALP. These comprised both individual case reports and larger series, with one study describing up to 26 patients. Collectively, these reports encompass over 100 patients, though the level of clinical detail varied across studies. Table 1 summarises the key findings from these reports.

### Time to presentation

The interval from RALP to clinical presentation ranged from 1 month to 252 months (21 years). While the majority of patients presented within the first 12 months, delayed presentations were not uncommon, underscoring the potential for late-onset clip migration.

### Clinical presentation

The most commonly reported symptoms included lower urinary tract symptoms (LUTS) such as dysuria, frequency, urgency, incomplete voiding, and urinary retention. Other presentations included:

- Visible haematuria
- Recurrent urinary tract infections
- Bladder neck contracture
- Perineal or pelvic pain
- Rarely, spontaneous clip expulsion

### Stone formation

Bladder stone formation was observed in the majority of cases where imaging or cystoscopic findings were reported. This suggests that prolonged intravesical presence of migrated clips predisposes to encrustation. In a minority of cases, HOLCs were found without associated calculi, indicating that early detection may help prevent stone development.

### Site of migration

The most frequent migration sites included the bladder and vesicourethral anastomosis, with a smaller number of clips embedded in the bladder neck or urethra. Migrated clips were often associated with local inflammation, granulation tissue, or stricture formation.

### Management

Nearly all cases were treated with endoscopic management. Reported techniques included:

- Cystoscopic removal with forceps or graspers
- Holmium or thulium laser lithotripsy
- Cold-knife bladder neck incision (BNI)
- Scar tissue vaporisation or urethral dilation

## Case vignettes

Two representative cases from our institution are presented to illustrate the clinical spectrum of delayed Hem-o-Lok migration.

### Case 1

A 68-year-old man with a background of hypertension and previous appendectomy and tonsillectomy underwent robotic-assisted radical prostatectomy with left-sided nerve sparing in October 2016 for Gleason 3 + 4 (Grade Group 2) T1c prostate adenocarcinoma. His prostate-specific antigen (PSA) at diagnosis was 6.2 µg/L, and staging MRI confirmed organ-confined disease. Surgery was uneventful aside from an estimated blood loss of 600 ml. He experienced a small pelvic collection and ileus postoperatively, which resolved with conservative management. Final pathology showed Gleason 4 + 3, pT2c, with negative margins.

The patient was subsequently placed on PSA surveillance. By 2021, flexible cystoscopy revealed a 3–4 cm bladder stone, which was addressed in February 2023 by laser cystolithotripsy. Intraoperatively, the core of the bladder stone was found to be a Hem-o-Lok clip, and two additional clips were seen eroding into the bladder from the left base. One clip was free and removed with the stone; the others were partially embedded – one successfully lasered, the other not retrievable due to being flush with the bladder wall.

CT imaging showed further Hem-o-Lok clips in the membranous urethra, likely from erosion over time (Figure 2). The patient has persistent stress urinary incontinence, using up to 5 pads/day, especially when mobile, and occasionally wears a urosheath. He has no significant urgency or urge incontinence, and his PSA remains undetectable ( <0.1 µg/L).

**Figure 2.**
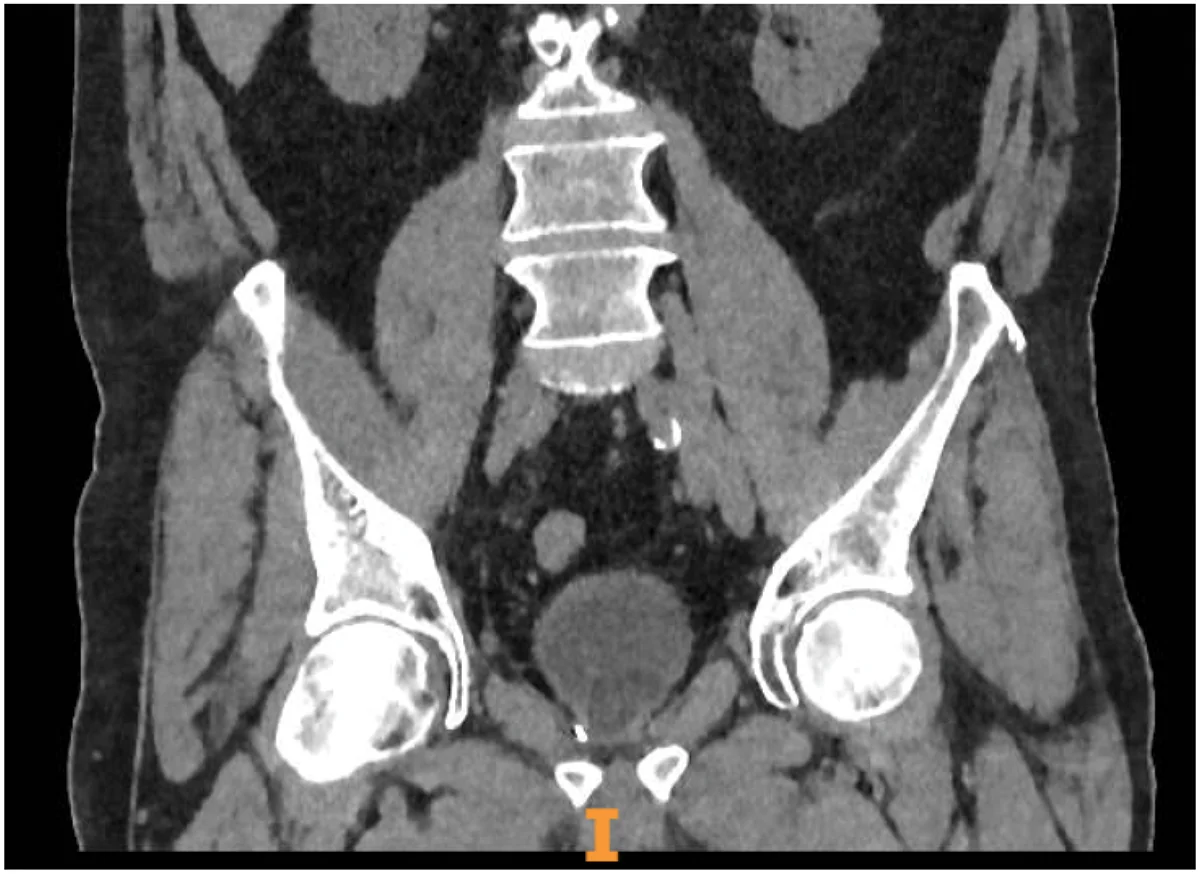
CT image demonstrating Hem-o-Lok® clips eroded into the membranous urethra.

After multidisciplinary discussion, the patient was recommended for rigid cystoscopy under general anaesthetic to attempt further clip extraction, with backup plans for open cystotomy if necessary. He is also being referred for specialist management of his incontinence, with consideration of bulking agents or artificial urinary sphincter. At the time of writing, he is listed for further surgery in late 2025.

### Case 2

A 64-year-old man was referred to the urology clinic in 2018 with mild lower urinary tract symptoms (LUTS) and a PSA level of 7.9 µg/L. He was otherwise fit and well, with no significant comorbidities, medications, or allergies. Prostate biopsies revealed high-volume Gleason 4 + 5 adenocarcinoma. MRI was not tolerated due to claustrophobia, but CT imaging suggested organ-confined disease with a suspicious right iliac lymph node, staging him as T3a N1 M0.

Following multidisciplinary discussion, the patient underwent robotic-assisted laparoscopic prostatectomy with extended pelvic lymph node dissection in December 2018. Histology confirmed high-risk, locally advanced prostate cancer. He subsequently received adjuvant radiotherapy to the prostate bed, completed in August 2019.

In 2022, the patient experienced biochemical recurrence and commenced androgen deprivation therapy. Disease progression prompted sequential systemic treatments with enzalutamide, then abiraterone (due to fatigue), and finally docetaxel chemotherapy, which he poorly tolerated. Despite this, he developed castrate-resistant metastatic disease by mid-2024, with symptomatic bone metastases and increasing frailty.

In April 2023, he presented with worsening urinary incontinence and penile pain and spontaneously passed a Hem-o-Lok clip per urethra during routine voiding (Figure 3). He had not undergone recent urological instrumentation. Interestingly, he reported an improvement in urinary symptoms following the event, including reduced incontinence. An urgent flexible cystoscopy was scheduled to evaluate for retained foreign material or erosion into the urinary tract. This investigation was pending at the time of reporting.

**Figure 3.**
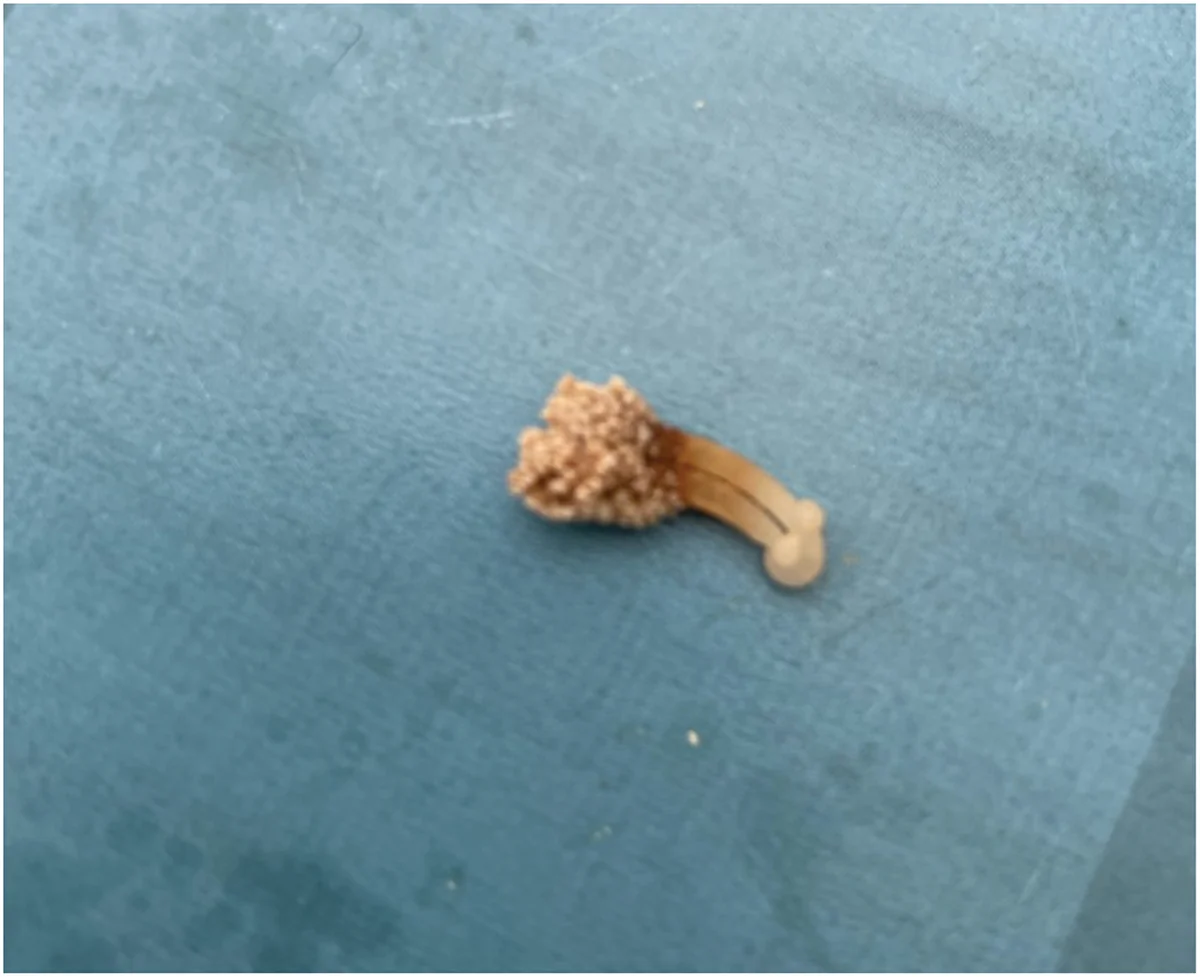
Spontaneously passed Hem-o-Lok clip with calcifications.

## Discussion

Hem-o-Lok clip migration is an uncommon but clinically significant complication following robot-assisted prostatectomy. As robotic techniques become increasingly standard in prostate cancer surgery, awareness of this delayed and often under-recognized issue is critical for long-term follow-up. Our case series and literature review highlight the complexity and clinical significance of HOLC migration following robotic prostatectomy. The two cases presented demonstrate the spectrum of presentations, from delayed stone formation requiring multiple interventions to spontaneous clip expulsion with symptom improvement.

### Incidence and timing

The reported incidence of HOLC migration varies considerably across studies, with figures ranging from 0.23% to 3.8%. For example, a recent single-centre study reported a 3.8% incidence among 682 RALP patients, while others have identified rates closer to 1–2% [17,22,25]. Although many cases present within the first year, migration can be delayed, sometimes significantly with cases being reported up to 11 years post-operatively [29]. Our literature review identified 89 cases across 24 studies, with a median time to presentation of 16.5 months (range 1–132 months). This wide range suggests that HOLC migration can occur early due to technical factors during surgery or late due to erosion and inflammatory processes [25].

### Pathophysiology and risk factors

Although the precise mechanism of Hem-o-Lok clip migration remains unclear, several contributory factors have been proposed. Poor or delayed healing at the vesicourethral anastomosis (VUA), often due to ischemia, necrosis, urine leakage, or chronic inflammation, may enable gradual erosion and clip migration into the urinary tract [17,25]. Clip placement too close to the anastomosis, particularly on adjacent structures like the vasa deferens or seminal vesicles, increases the risk, as does improper clip application or failure to retrieve loose clips [9,11,14,18]. Electrocautery near the VUA may exacerbate tissue devitalisation, while excessive intraoperative bleeding and pelvic haematoma formation may disrupt healing [11,25,32].

Patient-specific factors such as prolonged hospital stay, higher transfusion requirements, previous radiotherapy, may impair tissue recovery and increase migration risk. Turini et al. (2016) identified prolonged hospital stay and greater intraoperative blood transfusion requirements as significant predictors of subsequent clip migration; they hypothesised that delayed pelvic bleeding and haematoma formation may disrupt the posterior anastomosis, enabling clip migration into the bladder [22].

Radiotherapy may contribute to HOLC migration by impairing healing at the

VUA. Yu et al. (2015) reported that two of six patients with clip migration had received radiotherapy, suggesting a possible link between radiation-induced tissue fragility and migration risk [17]. Similarly, Bientinesi et al. (2015) described a patient who developed urinary leakage and underwent postoperative pelvic radiotherapy, hypothesising that this sequence may have favoured clip erosion into the anastomotic site [18]. This is supported by Zhu et al. (2021) who reported that patients with clip migration had significantly higher rates of urine leakage (15% vs. 6%, *p* = 0.046) and anastomotic stenosis (54% vs. 5%, *p* = 0.000) compared to those without migration [25].

### Clinical presentation

The clinic presentation of HOLC migration is variable and at times, nonspecific. LUTS are the most common presentation, including dysuria, frequency, urgency, and incomplete voiding with haematuria also a being common occurrence.

Yu et al. (2015) proposed a classification system of Hem-o-Lok migrations (Figure 4) corresponding to presenting symptoms [17].

**Figure 4.**
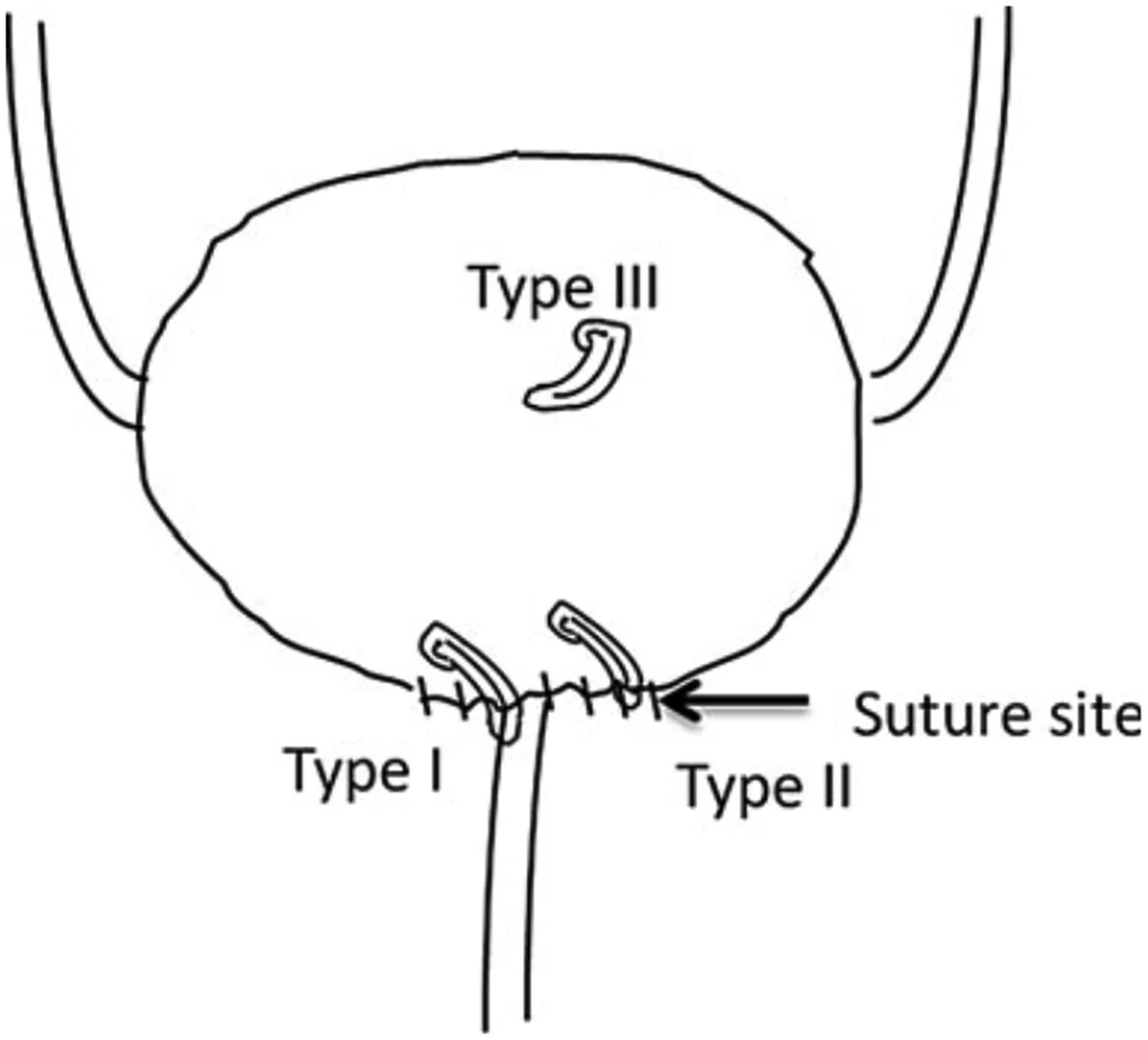
Illustrations of the three types of Hem-o-Lok clip migrations by Yu et al. [17].

- Type I: Erosion at the anastomosis causing obstructive LUTS and bladder neck contracture.
- Type II: Later erosion with stone formation, without acute obstruction.
- Type III: Early, spontaneous passage of loose, unretrieved clips.

Case 1 May represent a late Type III presentation, with spontaneous clip passage and no recent surgical insult. Case 2 aligns with Type II, where clip erosion resulted in a large bladder stone and embedded foreign material, likely contributing to recurrent UTIs and persistent incontinence.

### Diagnosis and management

Diagnosis of HOLC migration requires a high index of suspicion in patients presenting with LUTS after robotic prostatectomy. Flexible cystoscopy is the gold standard for diagnosis, allowing direct visualization of migrated clips and assessment of associated complications such as bladder neck contracture or stone formation. Cross-sectional imaging with CT or MRI can be valuable for identifying clips not visible on cystoscopy and assessing the extent of migration. In Case 2, clips were identified both intraoperatively and via CT at the membranous urethra, supporting the value of imaging when clip migration is suspected.

Management of HOLC migration can be variable. Cystoscopic removal is the preferred approach for accessible clips, with various techniques employed including forceps extraction, holmium laser lithotripsy for associated stones, and cold-knife incision for bladder neck contracture. In cases with extensive stone formation or multiple embedded clips, as in our first case, multiple procedures may be required. Open surgical removal may be necessary for clips that cannot be safely accessed endoscopically, as in consideration of our second case.

A structured management algorithm for post-RARP LUTS has been proposed by Singh et al. (2023) [32], incorporating imaging, cystoscopy, and tailored endoscopic treatment. TRUS or CT can identify intravesical clips, with subsequent cystoscopy used to assess for bladder neck contracture, stone formation, or embedded clips. Treatment options include cold knife incision, laser lithotripsy, and clip extraction. Re-cystoscopy is advised if symptoms persist despite catheterisation and follow-up (see Figure 5).

**Figure 5.**
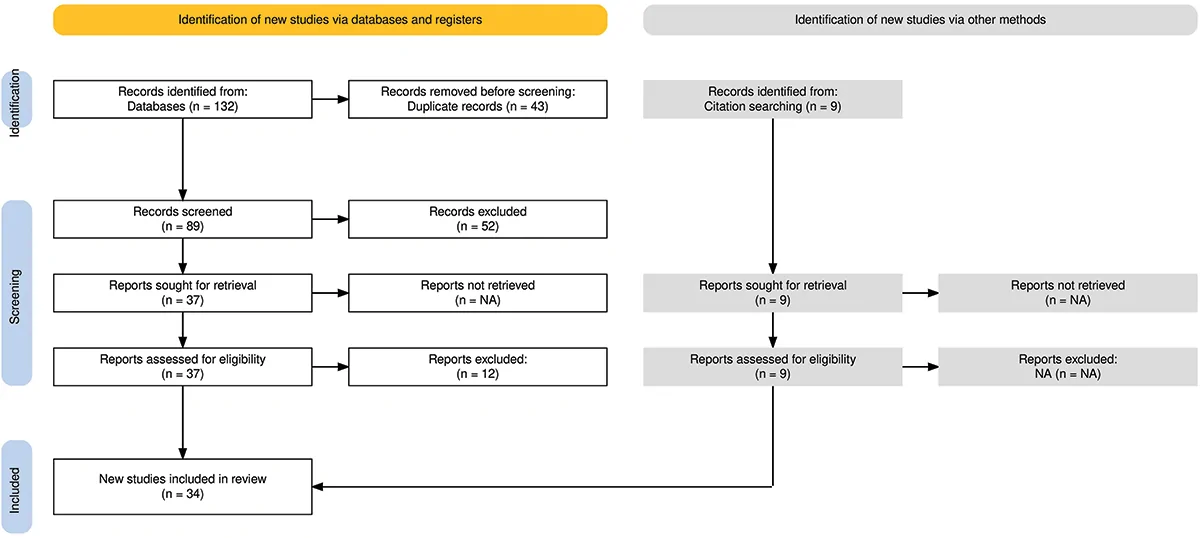
Algorithm for management of lower urinary tract symptoms presenting post RALP by Sing et al [32].

### Preventive strategies

Prevention of HOLC migration is paramount and involves several surgical principles. These centre on limiting clip use near the VUA, refining dissection technique, and adopting alternative haemostatic methods.

Clip use should be minimised, with clips confined to the proximal prostatic pedicles and avoided near the VUA, seminal vesicles, or vasa deferentia [1,9,25,28]. Appropriate clip sizing, secure application, and removal of loose clips are essential [32].

Electrocautery near the apex and bladder neck should be limited. Cold dissection with scissors and precise energy use can reduce devitalisation and improve healing [25].

Alternatives to clips include suture ligation, vessel-sealing systems (e.g. LigaSure), and bioadhesives [38–40]. These methods avoid foreign body risks and may improve operative outcomes, especially in nerve-sparing or complex cases.

Absorbable haemostatic options, such as polydioxanone-based absorbable clips (e.g. Absolok®), may further reduce long-term foreign body risk and represent a viable alternative in selected cases. Comparative data from minimally invasive renal surgery demonstrate equivalent perioperative and complication outcomes between absorbable and non-absorbable polymer clips, with the added advantage of clip resorption on follow-up imaging, thereby potentially mitigating risks of long-term erosion or migration [41].

Finally, clinician awareness is vital. In patients with post-RALP LUTS, recurrent UTIs, or haematuria, clip migration should be considered. Prompt imaging and cystoscopy can enable early detection and intervention.

### Limitations

This review is limited by the small number of cases presented, which restricts the ability to generalise findings or establish causation. As a retrospective case series, it may also be subject to incomplete documentation and reporting bias. These limitations underscore the need for larger, prospective studies to better define the incidence, risk factors, and optimal management strategies for Hem-o-Lok clip migration.

Although Jaeger et al. (2015) did not stratify cases by surgical approach, it was included due to the detailed characterisation of clip migration patterns and management strategies, which are mechanistically applicable across prostatectomy technique [19].

## Conclusion

Hem-o-Lok clip migration following robotic-assisted radical prostatectomy, though rare, represents a clinically important complication with potential for significant patient morbidity. Presentations are varied and may occur many years after the initial surgery. Our case series illustrates both the diagnostic challenges and therapeutic considerations in managing these patients.

A comprehensive review of the literature highlights key patterns of presentation, common risk factors, and evolving strategies for prevention and management. Surgeons should remain vigilant, particularly in patients with persistent or unexplained urinary symptoms post-RALP.

Preventive measures, such as minimising clip use near the vesicourethral anastomosis and adopting alternative haemostatic techniques, may reduce incidence. Prompt recognition and appropriate intervention remain essential to mitigating long-term sequelae and improving patient outcomes.

## Key learning points

- Clip migration can present months to decades post-RALP with LUTS/haematuria ± stones.
- Cystoscopy is diagnostic; endoscopic extraction is usually definitive.
- Risk factors: anastomotic devitalization/urine leak, clips near VUA, prior radiotherapy.
- Prevention: minimize clips near VUA; retrieve loose clips; use careful energy; consider suture/vessel-sealer alternatives.
